# Gel Volume Near the Critical Point of Binary Mixture Isobutyric Acid–Water

**DOI:** 10.3390/gels6030030

**Published:** 2020-09-12

**Authors:** Takao Yamamoto, Motoki Noguchi, Yasuyuki Maki, Toshiaki Dobashi

**Affiliations:** 1 Division of Pure and Applied Science, Graduate School of Science and Technology, Gunma University, Kiryu, Gunma 376-8515, Japan; 2 Division of Molecular Science, Graduate School of Science and Technology, Gunma University, Kiryu, Gunma 376-8515, Japan; mot.ngc@gmail.com (M.N.); maki@chem.kyushu-univ.jp (Y.M.); dobashi@gunma-u.ac.jp (T.D.)

**Keywords:** gel volume, UCST, binary mixture, Ising model, thermodynamic description, critical phenomena

## Abstract

The volume of a cylindrical polyacrylamide gel was measured when immersed in a binary mixture of isobutyric acid–water at different temperatures and weight fractions of isobutyric acid. Near the upper critical solution temperature of the binary mixture, the curve for gel volume vs. isobutyric acid weight fraction has a shoulder or a peak near the critical weight fraction. On the other hand, in a region away from the critical temperature, the gel volume decreased monotonically with increasing isobutyric acid weight fraction. The cloud point temperature of the binary mixture inside the gel was lower than that outside the gel. Thermodynamic description for the gel in the critical mixture is derived on the basis of the Ising model. By the description, the experimental results are explained consistently. The theoretical analysis shows that the shoulder and the peak appearing in the swelling behavior of the gel are respectively induced by the criticalities of the binary mixture outside and inside the gel. It also shows that the cloud point temperature lowering of the binary mixture inside the gel is attributed to the effective enhancement of the temperature of the binary mixture inside the gel induced by the presence of the gel polymer.

## 1. Introduction

Polymer gels are a three-dimensional network swollen in a solvent. They shrink or swell in response to the change in solvent property due to the modification of inter- and intramolecular segment–segment interactions. In mixed solvents (binary mixtures), a synergetic solvent power should be considered to discuss the conformation of polymer chains and the gel volume. As a binary mixture approaches its critical point, the concentration fluctuations grow, and finally, the size of the spatially inhomogeneous domains reaches the order of the pore size of the gel network. The large critical concentration fluctuations significantly affect the gel volume.

For a single polymer molecule in a critical mixture, de Gennes predicted that the polymer molecule first shrinks and then swells as the mixture approaches its critical point [1,2]. Many experimental and simulation studies on such a system have been carried out [3,4,5,6], and de Gennes’ prediction was confirmed by the results of both studies [7,8]. In the polymer networks in binary mixtures, the microscopic changes in the size of polymer chains are integrated into macroscopic volume changes. The changes are easily observed and analyzed without any ad hoc assumptions which are often associated with microscopic measurements.

For a polyacrylamide gel, an experimental study has recently been carried out and reported which focuses on the volume phase transition in the binary mixture 2-buthoxyethanol in water [9]. The study showed that the volume of the polyacrylamide gel near the lower critical solution temperature (LCST) $T_{\mathrm{lc}}$ of the binary mixture relative to that away from $T_{\mathrm{lc}}$ has an extremum around the critical composition, suggesting an effect of critical fluctuations. In this type of system that has LCST, the mechanism of phase behavior is rather complex due to interactions such as hydrogen bondings or zwitterionic interactions; thus, the quantitative comparison between theoretical and experimental results may be complex.

Critical fluctuations associated with the upper critical solution temperature (UCST) are more commonly observed even for simpler hydrophobic systems. In this study, we immersed a polyacrylamide gel in the isobutyric acid–water system whose critical phenomena near the critical point (critical temperature *T*_c_ = 26.4 °C and critical weight fraction of isobutyric acid *W*_c_ = 0.388) have been most intensively studied [10,11], and measured the gel volume as a function of the temperature and weight fraction of isobutyric acid in the solution. The plot for gel volume vs. weight fraction of isobutyric acid exhibited a shoulder near the critical temperature or a peak quite near, and just on, the critical temperature.

To discuss the effect of critical concentration fluctuations on the gel volume and explain the results for the above system observed, we attempted to derive a thermodynamic description of the system consisting of a gel in a critical binary mixture on the basis of the Ising-type lattice model. By paying attention to only the solution part, we construct a general theory independent of the details of the gel polymer. The gel swelling behavior is discussed from the viewpoint of the phase coexistence between the binary mixtures inside and outside the gel. The chemical potential balance between the two binary mixtures determines the gel volume. The chemical potential is expressed in terms of the external magnetic field of the Ising model. Then, the relationship between the gel volume and the weight fraction of the solution is expressed in terms of the relationship between the magnetization and the external magnetic field in the Ising model. The slope of the plot for the relationship between the gel volume and the weight fraction is expressed in terms of magnetic susceptibility. Strong fluctuations near the critical point induce anomaly growth of the susceptibility. We show that the anomaly of the susceptibility induces the characteristic behaviors, the presence of the shoulder and the peak in the plot of the gel volume near the critical point. We then develop the theory of the critical behavior of gel swelling.

## 2. Experimental Results

**Figure 1:**
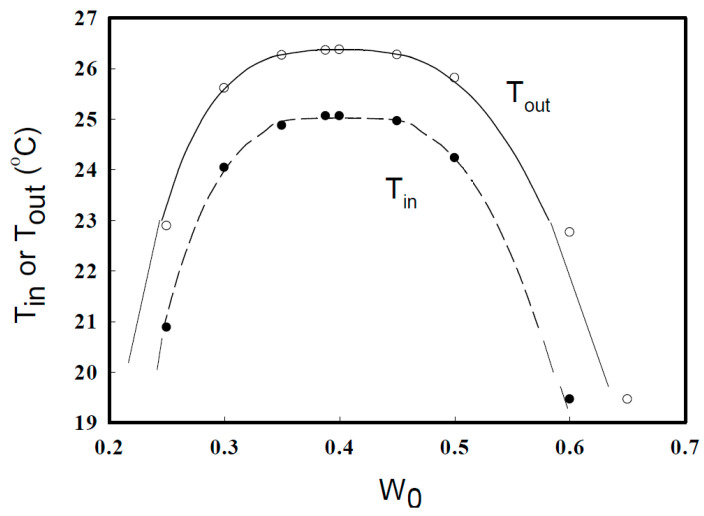
Temperatures at which the binary mixture of isobutyric acid–water with the weight fraction *W*_0_ inside (●) and outside (○) of the polyacrylamide gel becomes turbid upon cooling the gel in a binary mixture bath.

**Figure 2:**
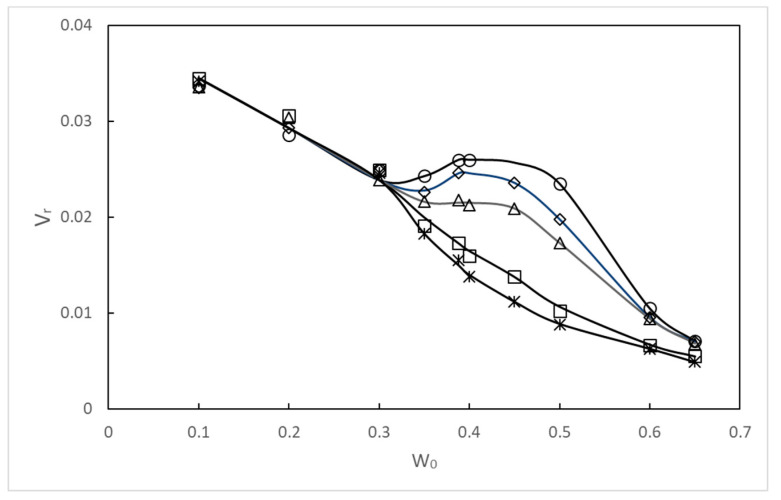
Reduced volume *V*r of polyacrylamide gel immersed in the binary mixture isobutyric acid–water at different weight fractions of isobutyric acid *W*_0_ and temperatures of 26.4 °C (○), 27.0 °C (◇), 28.0 °C (△), 35.0 °C (□), and 45.0 °C (*).

Figure 1 shows temperatures at which the binary mixture becomes turbid inside and outside of the gel (*T*_in_ and *T*_out_, respectively) as a function of the weight fraction *W*_0_ of isobutyric acid in the binary mixture upon cooling the gel in the mixture bath. Since the weight of the polymer in the gel is negligible compared to that of the immersing binary mixture, the plot of *T*_out_ vs. *W*_0_ is regarded as the cloud point curve of the binary mixture. The maximum temperature in the cloud point curve of the binary solution outside the gel was 26.4 °C, which agrees with the critical temperature determined for the coexistence curve [10], since the coexistence curve for two-component systems is identical to the cloud point curve. The condition inside the gel was much different from that outside the gel. At the temperature *T*_out_ when the binary mixture outside the gel became turbid, the mixture inside the gel remained transparent, i.e., *T*_in_ is lower than *T*_out_. At *T* < *T*_out_, the binary mixture separated into two phases and the gel was immersed in the lower (heavier) water-rich phase. Therefore, the curve for *T*_in_ should be regarded as an apparent cloud point curve. Figure 2 shows the ratio of the gel volume at different *W*_0_ values and temperatures to that at *W*_0_ = 0 and at UCST (26.4 °C), *V*_r_ as a function of *W*_0_. The gel volume decreases monotonically with increasing *W*_0_ except near the critical point, since water is a much better solvent for acrylamide gels than isobutyric acid. The gel volume in the binary mixture at a large *W*_0_ is as small as 1/200–1/30 of that in pure water. However, note that the degree of change in gel volume strongly depends on the cross-linking density of the gel. In this experiment, the amount of cross-linking reagents used to prepare gels was carefully determined so that the gels exhibit a large volume change by varying temperature and *W*_0_, and still have enough mechanical strength to be handled easily, by trial and error. In contrast, near the critical temperature, the curve *V*_r_ vs. *W*_0_ has a moderate shoulder or a peak near the critical weight fraction of isobutyric acid. The behavior of the gel volume similar to that of the present one has also been observed in LCST systems [9]. Therefore, this characteristic behavior could be attributed to the same mechanism associated with the critical phenomena. The Young’s modulus *E* of dilute gels is related to the number of monomers between neighboring cross-linking points *N*_c_ as *E* ~ *ckT*/*N*_c_ [12], where *c* is the number concentration of monomers in the gel, *k* the Boltzmann constant, and *T* the absolute temperature. From this equation, the distance between cross-linking points $L_{c}\sim {\left(\frac{kT}{E}\right)}^{1/3}$ was estimated to be 11 nm from the observed *E* for gels prepared by immersing them in pure water at 26.4 °C. This value is comparable to the correlation length around several degrees above from the critical point for the binary mixture isobutyric acid–water [13].

## 3. Theoretical Consideration

### 3.1. Lattice Model and Free Energy

Let us propose a lattice model for the polymer gel immersed in the binary mixture. The binary mixture in which the polymer gel is immersed is divided into a simple cubic lattice of L×L×L. A site on the lattice is denoted by $\vec{r}=\left(x,y,z\right)$, where x, y, and z are positive integer values, namely, x, y, z=1,2,⋯,L. The lattice is occupied by one of the three types of molecules, which are the solvent molecule, the solute molecule, and the segment of the polymer molecules in the gel. In terms of the “spin” variable $S\left(\vec{r}\right)$, the occupation is designated as:

(1) $$S\left(\vec{r}\right)=\{\begin{array}{cc} 1 & \mathrm{occupied}\;\mathrm{by}\;\mathrm{the}\;\mathrm{solvent}\\ -1 & \mathrm{occupied}\;\mathrm{by}\;\mathrm{the}\;\mathrm{solute}\\ 0 & \mathrm{occupied}\;\mathrm{by}\;\mathrm{the}\;\mathrm{segment} \end{array}$$

The gel–solution interaction is expressed by the spin-1 Ising model [14,15,16], whose Hamiltonian is given by

(2) $$H_{\mathrm{SI}}=H_{S}+H_{I},$$

(3) $$H_{S}=-K\underset{\langle \vec{r},{\vec{r}}^{\prime }\rangle }{\sum }S\left(\vec{r}\right)S\left({\vec{r}}^{\prime }\right)-h\underset{\vec{r}}{\sum }S\left(\vec{r}\right),\;$$

(4) $$H_{I}=-J\underset{\langle \vec{r},{\vec{r}}^{\prime }\rangle }{\sum }\left[S\left(\vec{r}\right)\left(1-S^{2}\left({\vec{r}}^{\prime }\right)\right)+S\left({\vec{r}}^{\prime }\right)\left(1-S^{2}\left(\vec{r}\right)\right)\right]-J_{1}\underset{\langle \vec{r},{\vec{r}}^{\prime }\rangle }{\sum }{\left[S^{2}\left(\vec{r}\right)-S^{2}\left({\vec{r}}^{\prime }\right)\right]}^{2},\;$$

where the positive parameter K denotes the interaction energy between the solvent and the solute molecules, and J and $J_{1}$ respectively denote the interaction energies between the segment and the solvent and between the segment and the solute. The parameter h is the “external field” and is a linear function of the difference between the chemical potentials of the solute molecules and the solvent molecules. The symbol $\langle \vec{r},{\vec{r}}^{\prime }\rangle$ denotes a nearest neighbor lattice-site pair. Note that only the interactions between the nearest neighbor lattice sites are taken into account in the above Hamiltonian.

Let the number of the polymer segments in the gel immersed in the binary solution be N and the lattice site occupied by the i-th segment (i=1,2,⋯,N) be denoted by ${\vec{R}}_{i}$. The Hamiltonian for the polymer chains composing the gel is a function of the segment lattice site ${\vec{R}}_{i}$. Let the Hamiltonian be denoted by $H_{G}\left(\left\{{\vec{R}}_{i}\right\}\right)$. Note that the constraint $S\left({\vec{R}}_{i}\right)=0$ is imposed on the lattice occupation state of the segment lattice site.

The partition function for the binary mixture–gel system is given by:

(5) $$Z\left(h,N,\Omega \right)=\underset{\left\{{\vec{R}}_{i}\neq {\vec{R}}_{j}\right\}}{\sum }e^{-\beta H_{G}\left(\left\{{\vec{R}}_{i}\right\}\right)}\underset{{\left\{S\left(\vec{r}\right)=0,\pm 1\right\}}_{\vec{r}}}{\sum }\left(\overset{N}{\underset{i=1}{\prod }}\delta_{S\left({\vec{R}}_{i}\right),0}\right)e^{-\beta H_{\mathrm{SI}}}\;=\underset{\left\{{\vec{R}}_{i}\neq {\vec{R}}_{j}\right\}}{\sum }e^{-\beta [H_{G}\left(\left\{{\vec{R}}_{i}\right\}\right)+F_{\mathrm{SI}}\left(\left\{{\vec{R}}_{i}\right\}\right)]},\;$$

where $\beta ={\left(k_{B}T\right)}^{-1}$ ($k_{B}$ is the Boltzmann constant and T is the absolute temperature) and $\Omega =L^{3}$ is the total lattice number. The quantity $F_{\mathrm{SI}}\left(\left\{{\vec{R}}_{i}\right\}\right)$ given by:

(6) $$F_{\mathrm{SI}}\left(\left\{{\vec{R}}_{i}\right\}\right)=-k_{B}T\ln Z_{\mathrm{SI}}\left(\left\{{\vec{R}}_{i}\right\}\right)\;$$

with

(7) $$Z_{\mathrm{SI}}\left(\left\{{\vec{R}}_{i}\right\}\right)=\underset{{\left\{S\left(\vec{r}\right)=0,\pm 1\right\}}_{\vec{r}}}{\sum }\left(\overset{N}{\underset{i=1}{\prod }}\delta_{S\left({\vec{R}}_{i}\right),0}\right)e^{-\beta H_{\mathrm{SI}}}\;$$

is the free energy of the binary solution in which the given lattice sites ${\left\{{\vec{R}}_{i}\right\}}_{i=1}^{N}$ are occupied by the polymer segments in the gel.

Let us ignore the dependence of $Z_{\mathrm{SI}}\left(\left\{{\vec{R}}_{i}\right\}\right)$ on the distribution of the lattice sites occupied by the polymer segments in the gel; we adopt the following approximation:

(8) $$Z_{\mathrm{SI}}\left(\left\{{\vec{R}}_{i}\right\}\right)≃{\bar{Z}}_{\mathrm{SI}}\left(h,N,\Omega \right)\equiv \underset{{\left\{S\left(\vec{r}\right)=0,\pm 1\right\}}_{\vec{r}}}{\sum }\delta (\underset{\vec{r}}{\sum }S^{2}\left(\vec{r}\right)|\Omega -N)e^{-\beta H_{\mathrm{SI}}}$$

In the above, δ(n|m) stands for the Kronecker delta; $\delta \left(n|m\right)\equiv \delta_{n,m}$. The approximation gives an approximated expression of Z(h,N,Ω) as:

(9) $$Z\left(h,N,\Omega \right)≃\bar{Z}\left(h,N,\Omega \right)\equiv {\bar{Z}}_{G}\left(N,\Omega \right){\bar{Z}}_{\mathrm{SI}}\left(h,N,\Omega \right)\;$$

with

(10) $${\bar{Z}}_{G}\left(N,\Omega \right)=\underset{\left\{{\vec{R}}_{i}\neq {\vec{R}}_{j}\right\}}{\sum }e^{-\beta H_{G}\left(\left\{{\vec{R}}_{i}\right\}\right)}\;$$

Therefore, the total free energy of the system is given by:

(11) $$F\left(h,N,\Omega \right)=F_{G}\left(N,\Omega \right)+F_{\mathrm{SI}}\left(h,N,\Omega \right)\;$$

where

(12) $$F_{G}\left(N,\Omega \right)=-k_{B}T\ln{\bar{Z}}_{G}\left(N,\Omega \right)\;$$

is the free energy of the gel and

(13) $$F_{\mathrm{SI}}\left(h,N,\Omega \right)=-k_{B}T\ln{\bar{Z}}_{\mathrm{SI}}\left(h,N,\Omega \right)\;$$

is the free energy of the binary solution having the interaction with the polymer segments of the gel. Note that the gel free energy $F_{G}$ is independent of h. The free energies per lattice site are also given as:

(14) $$\tilde{f}\left(h,\phi \right)=\frac{1}{\Omega }F\left(h,N,\Omega \right)=f_{G}\left(\phi \right)+{\tilde{f}}_{\mathrm{SI}}\left(h,\phi \right)\;$$

(15) $$f_{G}\left(\phi \right)=\frac{1}{\Omega }F_{G}\left(N,\Omega \right)\;$$

(16) $${\tilde{f}}_{\mathrm{SI}}\left(h,\phi \right)=\frac{1}{\Omega }F_{\mathrm{SI}}\left(h,N,\Omega \right)\;,\;$$

where ϕ is the volume fraction of the polymer segment in the gel defined by:

(17) $$\phi =\frac{N}{\Omega }\;.\;$$

From Equation (16) with Equation (8), we have

(18) $$-\frac{\partial {\tilde{f}}_{\mathrm{SI}}}{\partial h}=\frac{1}{\Omega }\langle \underset{\vec{r}}{\sum }S\left(\vec{r}\right)\rangle =\langle S\left(\vec{r}\right)\rangle \equiv Q\;,\;$$

where 〈⋯〉 denotes the thermal average. The quantity Q stands for the order parameter of the spin-1 Ising model. Denoting the numbers of the solvent and the solute as $n_{0}$ and $n_{1}$, we have

(19) $$n_{0}+n_{1}+N=\Omega \;$$

and

(20) $$Q=\frac{n_{1}-n_{0}}{\Omega }\;,\;$$

respectively. In terms of ϕ and Q, $n_{0}$ and $n_{1}$ are written as $n_{0}=\frac{1}{2}\left(1-Q-\phi \right)\Omega$ and $n_{1}=\frac{1}{2}\left(1+Q-\phi \right)\Omega$.

By Legendre transformation, we obtain the Helmholtz free energy whose arguments are Q and ϕ by

(21) $$f_{\mathrm{SI}}\left(Q,\phi \right)={\tilde{f}}_{\mathrm{SI}}\left(h,\phi \right)+Qh\;,\;$$

where Q is defined by Equation (18).

The Helmholtz free energy per lattice site of the system is given by:

(22) $$f\left(Q,\phi \right)=f_{G}\left(\phi \right)+f_{\mathrm{SI}}\left(Q,\phi \right)\;.\;$$

In terms of f(Q,ϕ) or $f_{\mathrm{SI}}\left(Q,\phi \right)$, h is obtained as:

(23) $$h=\frac{\partial f\left(Q,\phi \right)}{\partial Q}=\frac{\partial f_{\mathrm{SI}}\left(Q,\phi \right)}{\partial Q}\;.\;$$

In terms of $f_{\mathrm{SI}}\left(Q,\phi \right)$, the Gibbs free energy is given by [17]:

(24) $$G\left(N,n_{0},n_{1}\right)=\Omega f\left(Q,\phi \right)+P\Omega v_{C}\;,\;$$

where P is the pressure and $v_{C}$ is the volume of a lattice.

### 3.2. Phase Equilibrium between Pure Binary Mixture and Gel Swollen by Binary Mixture

In the system of the gel immersed in the binary mixture, the gel swollen by the binary mixture (i.e., the gel phase) and the pure binary mixture (i.e., the binary mixture phase) coexist. The condition under which the gel phase and the binary mixture phase coexist requires that the chemical potentials of the solvent and solute molecules should be balanced between the gel phase and the binary mixture phase. The chemical potentials of the solvent and solute molecules are respectively given by the following [17]:

(25) $$\begin{array}{ll} \mu_{0} & =G\left(N,n_{0}+1,n_{1}\right)-G\left(N,n_{0},n_{1}\right)≃{\left(\frac{\partial G}{\partial n_{0}}\right)}_{N,n_{1}}\\ & =f\left(Q,\phi \right)-\left(1+Q\right){\left(\frac{\partial f}{\partial Q}\right)}_{\phi }-\phi {\left(\frac{\partial f}{\partial \phi }\right)}_{Q}+Pv_{C}\; \end{array}$$

(26) $$\begin{array}{ll} \mu_{1} & =G\left(N,n_{0},n_{1}+1\right)-G\left(N,n_{0},n_{1}\right)≃{\left(\frac{\partial G}{\partial n_{1}}\right)}_{N,n_{0}}\\ & =f\left(Q,\phi \right)+\left(1-Q\right){\left(\frac{\partial f}{\partial Q}\right)}_{\phi }-\phi {\left(\frac{\partial f}{\partial \phi }\right)}_{Q}+Pv_{C}\; \end{array}$$

The difference between the two chemical potentials is given by:

(27) $$\Delta \mu \left(Q,\phi \right)=\mu_{1}\left(Q,\phi \right)-\mu_{0}\left(Q,\phi \right)=2\frac{\partial f\left(Q,\phi \right)}{\partial Q}=2\frac{\partial f_{\mathrm{SI}}\left(Q,\phi \right)}{\partial Q}\;$$

When $\mu_{0}$ and $\mu_{1}$ are balanced between the gel phase and the binary mixture phase, Δμ is also balanced. Therefore, denoting the order parameter value in the gel as Q=Q and that in the binary mixture phase by $Q=Q_{0}$, we have

(28) $$\Delta \mu \left(Q,\phi \right)=\Delta \mu \left(Q_{0},0\right)\;;\;$$

hence:

(29) $$\frac{\partial f_{\mathrm{SI}}\left(Q,\phi \right)}{\partial Q}=\frac{\partial f_{\mathrm{SI}}\left(Q_{0},0\right)}{\partial Q}\;.\;$$

Equation (28) shows the balance of the external field as a function of Q and , $h\left(Q,\phi \right)=\partial f_{\mathrm{SI}}\left(Q,\phi \right)/\partial Q$:

(30) $$h\left(Q,\phi \right)=h\left(Q_{0},0\right)\;.\;$$

The solute chemical potential balance $\mu_{1}\left(Q,\phi \right)=\mu_{1}\left(Q_{0},0\right)$ gives us the equation

(31) $$\begin{array}{l} f\left(Q,\phi \right)+\left(1-Q\right)h\left(Q,\phi \right)-\phi {\left(\frac{\partial f}{\partial \phi }\right)}_{Q}\\ =f\left(Q_{0},0\right)+\left(1-Q_{0}\right)h\left(Q_{0},0\right)\;. \end{array}$$

Using Equations (21) and (30), we rewrite Equation (31) as:

(32) $$\begin{array}{r} f_{G}\left(\phi \right)-\phi \frac{df_{G}\left(\phi \right)}{d\phi }-\phi {\left(\frac{\partial {\tilde{f}}_{\mathrm{SI}}\left(h\left(Q_{0},0\right),\phi \right)}{\partial \phi }\right)}_{h\left(Q_{0},0\right)}\\ ={\tilde{f}}_{\mathrm{SI}}\left(h\left(Q_{0},0\right),0\right)-{\tilde{f}}_{\mathrm{SI}}\left(h\left(Q_{0},0\right),\phi \right)\;,\; \end{array}$$

where we choose the origin of the gel free energy $f_{G}\left(\phi \right)$ such that $f_{G}\left(0\right)=0$. From Equation (32), the volume fraction of the polymer segment in the gel (ϕ) is obtained as a function of $Q_{0}$.

(33) $$\phi =\phi_{\mathrm{eq}}\left(Q_{0}\right)\;$$

Denoting the value of the order parameter in the gel phase as $Q_{\mathrm{eq}}$ and using the expression $\phi_{\mathrm{eq}}$ instead of the expression ϕ, we rewrite Equation (30) as:

(34) $$h\left(Q_{\mathrm{eq}},\phi_{\mathrm{eq}}\left(Q_{0}\right)\right)=h\left(Q_{0},0\right)\;.\;$$

From the balanced Equation (34), we have the order parameter $Q_{\mathrm{eq}}$ of the gel phase as a function of $Q_{0}$:

(35) $$Q_{\mathrm{eq}}=Q_{\mathrm{eq}}\left(Q_{0}\right)\;$$

The quantity $Q_{0}$ stands for the composition of the binary mixture phase. Therefore, $\phi_{\mathrm{eq}}\left(Q_{0}\right)$ and $Q_{\mathrm{eq}}\left(Q_{0}\right)$ stand for the dependences of the gel volume fraction and the composition of the gel phase, respectively, on the composition of the binary mixture in which the gel is immersed.

### 3.3. Gel Volume Change and Critical Phenomena

Equation (32) must be satisfied by the function $\phi =\phi_{\mathrm{eq}}\left(Q_{0}\right).$

(36) $$\begin{array}{ll} f_{G}\left(\phi_{\mathrm{eq}}\left(Q_{0}\right)\right) & -\phi_{\mathrm{eq}}\left(Q_{0}\right)\frac{df_{G}\left(\phi_{\mathrm{eq}}\left(Q_{0}\right)\right)}{d\phi_{\mathrm{eq}}}\\ & -\phi_{\mathrm{eq}}\left(Q_{0}\right){\left(\frac{\partial {\tilde{f}}_{\mathrm{SI}}\left(h\left(Q_{0},0\right),\phi_{\mathrm{eq}}\left(Q_{0}\right)\right)}{\partial \phi_{\mathrm{eq}}}\right)}_{h\left(Q_{0},0\right)}\\ & ={\tilde{f}}_{\mathrm{SI}}\left(h\left(Q_{0},0\right),0\right)-{\tilde{f}}_{\mathrm{SI}}\left(h\left(Q_{0},0\right),\phi_{\mathrm{eq}}\left(Q_{0}\right)\right)\; \end{array}$$

Differentiating both sides of the above equation with respect to $Q_{0}$, we have:

(37) $$-\frac{d\phi_{\mathrm{eq}}}{dQ_{0}}\phi_{\mathrm{eq}}\left(f_{G}^{\prime \prime }+\frac{\partial^{2}{\tilde{f}}_{\mathrm{SI}}\left(h\left(Q_{0},0\right),\phi_{\mathrm{eq}}\left(Q_{0}\right)\right)}{\partial^{2}\phi_{\mathrm{eq}}}\right)\;=\frac{\partial h\left(Q_{0},0\right)}{\partial Q_{0}}\left(Q_{\mathrm{eq}}\left(Q_{0}\right)-Q_{0}+\phi_{\mathrm{eq}}\left(Q_{0}\right)\frac{\partial^{2}{\tilde{f}}_{\mathrm{SI}}\left(h\left(Q_{0},0\right),\phi_{\mathrm{eq}}\left(Q_{0}\right)\right)}{\partial h\partial \phi_{\mathrm{eq}}}\right)\;,$$

where $f_{G}^{\prime \prime }=d^{2}f_{G}\left(\phi_{\mathrm{eq}}\left(Q_{0}\right)\right)/d\phi_{\mathrm{eq}}^{2}$.

In terms of $\phi_{\mathrm{eq}}$, the volume of the gel $V_{G}$ is given by:

(38) $$V_{G}=\frac{Nv_{C}}{\phi_{\mathrm{eq}}}\;,\;$$

and the dependence of the gel volume on the composition of the binary mixture surrounding the gel is expressed as:

(39) $$\frac{dV_{G}}{dQ_{0}}=-\frac{Nv_{C}}{\phi_{\mathrm{eq}}^{2}}\frac{d\phi_{\mathrm{eq}}}{dQ_{0}}\;$$

Let us assume that the quantity

(40) $$A\left(Q_{0}\right)\equiv \phi_{\mathrm{eq}}^{2}\left(f_{G}^{\prime \prime }+\frac{\partial^{2}{\tilde{f}}_{\mathrm{SI}}\left(h\left(Q_{0},0\right),\phi_{\mathrm{eq}}\left(Q_{0}\right)\right)}{\partial^{2}\phi_{\mathrm{eq}}}\right)\;$$

does not vanish since the free energy $\tilde{f}\left(h,\phi \right)=f\left(Q,\phi \right)+hQ=f_{G}\left(\phi \right)+{\tilde{f}}_{\mathrm{SI}}\left(h,\phi \right)$ is expected not to be very sensitive to ϕ; the sign of the second derivative $\partial^{2}\tilde{f}\left(h,\phi \right)/\partial \phi^{2}$ does not alter and is positive in the reasonable parameter regions. Using Equation (37), the relation (39), and the expression (40), we have:

(41) $$\frac{dV_{G}}{dQ_{0}}=V_{G}\frac{\partial h\left(Q_{0},0\right)}{\partial Q_{0}}\frac{B\left(Q_{0}\right)}{A\left(Q_{0}\right)}\;,\;$$

where

(42) $$B\left(Q_{o}\right)=Q_{\mathrm{eq}}\left(Q_{0}\right)-Q_{0}+\phi_{\mathrm{eq}}\frac{\partial^{2}{\tilde{f}}_{\mathrm{SI}}\left(h\left(Q_{0},0\right),\phi_{\mathrm{eq}}\left(Q_{0}\right)\right)}{\partial h\partial \phi_{\mathrm{eq}}}\;.\;$$

The system with ϕ = 0 is the conventional Ising model in which the spin value $S\left(\vec{r}\right)$ takes +1 or −1. From the viewpoint of the Ising model, $\partial h\left(Q_{0},0\right)/\partial Q_{0}$ in Equation (41) is the inverse of the magnetic susceptibility $\chi_{m}.$

(43) $$\frac{\partial h\left(Q_{0},0\right)}{\partial Q_{0}}=\chi_{m}^{-1}\;$$

Hence, the volume change with respect to the composition of the binary mixture can be related to the “susceptibility” of the binary mixture as:

(44) $$\frac{dV_{G}}{dQ_{0}}=\frac{B\left(Q_{0}\right)}{A\left(Q_{0}\right)}\chi_{m}^{-1}\left(Q_{0}\right)\;.\;$$

At the critical temperature $T=T_{C}$, $\chi_{m}$ behaves as:

(45) $$\chi_{m}∼{\left|\Delta Q_{0}\right|}^{1-\delta }\;,\;$$

with the exponent δ≃4.8 for the three-dimensional Ising model [18,19], where $\Delta Q_{0}$ is the deviation of the composition from the critical composition $Q^{*}$ (for the lattice model introduced in the present article, $Q^{*}=0$) of the pure binary mixture.

(46) $$\Delta Q_{0}=Q_{0}-Q^{*}\;$$

Therefore, near the critical composition, the gel volume changes as:

(47) $$\frac{dV_{G}}{dQ_{0}}≃CV_{G}^{*}\chi_{m}^{-1}∼{\left|\Delta Q_{0}\right|}^{\delta -1}≃{\left|\Delta Q_{0}\right|}^{3.8}\;,\;$$

where $V_{G}^{*}=V_{G}\left(Q^{*}\right)$ is the gel volume at the critical point and $C=B\left(Q^{*}\right)/A\left(Q^{*}\right)$ is a constant. Equation (47) shows the “critical behavior” of gel swelling; the swelling function $V_{G}\left(Q_{0}\right)$ is singular at $Q_{0}=Q^{*}$. The critical behavior is characterized by the critical exponent of the pure binary mixture surrounding the gel. The critical composition $Q_{0}=Q^{*}$ is on the inflection point of the $V_{G}$–$Q_{0}$ curve, and the $V_{G}$–$Q_{0}$ curve is a straight line parallel to the $Q_{0}$-axis in a wide area around the inflection point.

Let us introduce a length scale characterizing the critical behavior of the gel swelling shown by Equation (47). Denoting the number of the cross-linking point in the gel by $n_{\mathrm{CL}}$, we can estimate the average distance $l_{\mathrm{CL}}$ between the neighboring cross-linking points at the critical composition as:

(48) $$l_{\mathrm{CL}}={\left(\frac{V_{G}^{*}}{n_{\mathrm{CL}}}\right)}^{\frac{1}{3}}\;.\;$$

In terms of $l_{\mathrm{CL}}$, the slope $dV_{G}/dQ_{0}\;$ of the $V_{G}$–$Q_{0}$ curve near the critical composition is rewritten as:

(49) $$\frac{dV_{G}}{dQ_{0}}≃v_{C}n_{\mathrm{CL}}{\left(\frac{l_{\mathrm{CL}}}{{\left(C^{-1}v_{C}\chi_{m}\right)}^{\frac{1}{3}}}\right)}^{3}=v_{C}n_{\mathrm{CL}}{\left(\frac{l_{\mathrm{CL}}}{l_{\mathrm{FL}}\left(\Delta Q_{0}\right)}\right)}^{3},\;$$

where

(50) $$l_{\mathrm{FL}}\left(\Delta Q_{0}\right)={\left(\frac{v_{C}}{C}\chi_{m}\right)}^{\frac{1}{3}},\;$$

is the length scale defined in terms of the susceptibility. The length $l_{\mathrm{FL}}$ becomes longer as the composition $Q_{0}$ approaches the critical component $Q^{*}$, and diverges as:

(51) $$l_{\mathrm{FL}}\left(\Delta Q_{0}\right)∼{\left|\Delta Q_{0}\right|}^{\frac{1-\delta }{3}\;}≃{\left|\Delta Q_{0}\right|}^{-1.3}\;$$

The divergence behavior of the length $l_{\mathrm{FL}}$ reflects the concentration fluctuation growth in the binary mixture. It is also a translation of the critical behavior of the gel swelling shown by Equation (47) in the language of length. When the length $l_{\mathrm{FL}}$ becomes much larger than the average distance $l_{\mathrm{CL}}$ between the cross-linking points, the slope $dV_{G}/dQ_{0}\;$ of the $V_{G}$–$Q_{0}$ curve vanishes and the gel volume becomes less sensitive to the composition of the binary mixture outside the gel. Although $l_{\mathrm{FL}}$ is different from the correlation length, $l_{\mathrm{FL}}$ expresses the magnitude of the concentration fluctuation in the language of length as the correlation length.

## 4. Discussion: Theoretical Analysis of Experimental Results

First, the relationship between the cloud point curves outside and inside the gel shown in Figure 1 is discussed on the basis of the lattice model Hamiltonian given by Equations (2)–(4). Let the spin-1 Ising spin variables $S\left(\vec{r}\right)$ be denoted as:

(52) $$S\left(\vec{r}\right)=\sigma \left(\vec{r}\right)\left(1-\rho \left(\vec{r}\right)\right)\;,\;$$

where $\sigma \left(\vec{r}\right)=\pm 1$ stands for the lattice site $\vec{r}$ being occupied by the solvent molecule when $\sigma \left(\vec{r}\right)=-1$ and being occupied by the solute molecule when $\sigma \left(\vec{r}\right)=1$, and $\rho \left(\vec{r}\right)$ stands for the occupation by the polymer segment ($\rho \left(\vec{r}\right)=1$ when the lattice site $\vec{r}$ is occupied by the polymer segment and $\rho \left(\vec{r}\right)=0$ when not occupied). Here, we adopt an approximation in which the lattice site is allowed to be occupied by the solution molecule and the polymer segment. In terms of σ and ρ, we have

(53) $$H_{S}=-K\underset{\langle \vec{r},{\vec{r}}^{\prime }\rangle }{\sum }\left(1-\rho \left(\vec{r}\right)\right)\left(1-\rho \left({\vec{r}}^{\prime }\right)\right)\sigma \left(\vec{r}\right)\sigma \left({\vec{r}}^{\prime }\right)\;-h\underset{\vec{r}}{\sum }\left(1-\rho \left(\vec{r}\right)\right)\sigma \left(\vec{r}\right),\;$$

and

(54) $$H_{I}=-J\underset{\langle \vec{r},{\vec{r}}^{\prime }\rangle }{\sum }\left[\left(1-\rho \left(\vec{r}\right)\right)\rho \left({\vec{r}}^{\prime }\right)\sigma \left(\vec{r}\right)+\left(1-\rho \left(\vec{r}\prime \right)\right)\rho \left(\vec{r}\right)\sigma \left(\vec{r}\prime \right)\right]\;-J_{1}\underset{\langle \vec{r},{\vec{r}}^{\prime }\rangle }{\sum }{\left[\rho \left(\vec{r}\right)-\rho \left({\vec{r}}^{\prime }\right)\right]}^{2}\;.\;$$

In the binary solution outside the gel, $\rho \left(\vec{r}\right)=0$ for all the lattice sites. Therefore, the Hamiltonian for the solution outside the gel is given by:

(55) $$H_{\mathrm{SI}}^{\mathrm{out}}=-K\underset{\langle \vec{r},{\vec{r}}^{\prime }\rangle }{\sum }\sigma \left(\vec{r}\right)\sigma \left({\vec{r}}^{\prime }\right)-h\underset{\vec{r}}{\sum }\sigma \left(\vec{r}\right)\;.\;$$

A simple mean field approximation, $\rho \left(\vec{r}\right)≃\phi_{\mathrm{eq}}$, is adopted for the binary solution inside the gel, and the Hamiltonian divided by $k_{B}T$ is given as

(56) $$\frac{H_{\mathrm{SI}}^{\mathrm{in}}}{k_{B}T}=\frac{-K\sum_{\langle \vec{r},{\vec{r}}^{\prime }\rangle }\sigma \left(\vec{r}\right)\sigma \left({\vec{r}}^{\prime }\right)-h_{\mathrm{eff}}\sum_{\vec{r}}\sigma \left(\vec{r}\right)}{k_{B}T_{\mathrm{eff}}}\;$$

with

(57) $$T_{\mathrm{eff}}=\frac{T}{{\left(1-\phi_{\mathrm{eq}}\right)}^{2}}\;$$

and

(58) $$h_{\mathrm{eff}}=\frac{h+z_{0}J\phi_{\mathrm{eq}}}{1-\phi_{\mathrm{eq}}}≃h+\left(h+z_{0}J\right)\phi_{\mathrm{eq}}\;.$$

In the above, $z_{0}$ is the number of the nearest neighbor lattice sites. Both the binary mixtures outside and inside the gel are expressed by the conventional Ising model, but the temperature and the external field are modified by the presence of polymer segments. The “effective” temperature $T_{\mathrm{eff}}$ of the binary mixture inside the gel is higher than the temperature T outside the gel. This means that the critical temperature $T_{c}^{\prime }$ of the binary mixture inside the gel is lower than the critical temperature $T_{c}$ outside the gel by the factor ${\left(1-\phi_{\mathrm{eq}}\right)}^{2}$. This result is consistent with the shift of the cloud point curve of the binary mixture inside the gel from that outside the gel downward, as shown in Figure 1. In the binary mixture inside the gel, the polymer segments block the interaction between the component molecules of the binary mixture, and the blocking decreases the interaction energy. The decrease in interaction energy is expressed by the increase in temperature. The interaction between the polymer segments and the component molecules of the binary mixture generates a microscopic external field acting on the component molecules of the binary mixture. Therefore, as shown by Equation (58), the external field is modified depending on $\phi_{\mathrm{eq}}$. From the viewpoint of the modified Ising expression for the binary mixture, the difference between the weight fractions $W_{\mathrm{eq}}$ of the inside solution and $W_{0}$ of the outside solution is induced by the modification of the external field.

Next, let us discuss the experimental results shown in Figure 2 on the basis of the theory developed in the previous section.

Figure 2 shows that the $V_{r}$–*W*_0_ curves monotonically decrease with increasing $W_{0}$ at high temperatures (T=35.0 ℃, 45.0 ℃), but at T=28.0 ℃, it has a wide shoulder around the “critical” weight fraction of isobutyric acid $W_{0}=W_{c}≃0.388$; $dV_{r}/dW_{0}≃0$ around $W_{0}=W_{c}$. On the basis of the theoretical result Equation (44), the reason for the presence of the wide shoulder can be explained as follows. Near the critical temperature, the susceptibility has large values (thus, $\chi_{m}^{-1}≃0$) around the critical composition. Therefore, in the wide region around the critical composition, $dV_{G}/dQ_{0}\approx 0$. Hence, the swelling curve $V_{G}=V_{G}(Q_{0}$) has a wide shoulder. The presence of the wide shoulder is a consequence of the critical phenomena of the binary mixture isobutyric acid–water. The range in which $dV_{r}/dW_{0}≃0$ is estimated is on the basis of concentration fluctuations. Near the critical point, long-range concentration fluctuations appear. As shown in the previous section, the range of the fluctuation is estimated by the length $l_{\mathrm{FL}}$. As the weight fraction $W_{0}$ approaches the critical weight fraction $W_{c}$, the length $l_{\mathrm{FL}}$ diverges as $l_{\mathrm{FL}}∼x_{m}^{\frac{1}{3}}∼{\left|W_{0}-W_{c}\right|}^{-1.3}$. When $l_{\mathrm{FL}}$ becomes longer than the distance of the cross-linking points $l_{\mathrm{CL}}≃{\left(0.02\right)}^{\frac{1}{3}}\times L_{c}≃3.0\;\mathrm{nm}$, the concentration fluctuations smear the difference between the affinities of the polymer (polyacrylamide) segment for the solvent (water) and solute (isobutyric acid); the gel volume is insensitive to the composition of the binary mixture. The wide shoulder reflects this insensitivity. The composition dependence of the gel volume appears when the fluctuation range becomes shorter than the distance between the cross-linking points away from the critical point.

At the temperature T=27.0 ℃, which is quite near the critical temperature, and at the critical temperature $T=T_{c}=$26.5 ℃, the $V_{r}$—*W*_0_ curve has a maximum at $W_{0}=W_{c}^{*}≃W_{c}$. In other words, at $W_{0}=W_{c}^{*}$, the sign of the slope of the $V_{r}—$*W* curve changes.

(59) $$\{\begin{array}{cc} \frac{dV_{r}}{dW_{0}}>0 & \mathrm{for}\;W_{0}<W_{c}^{*}\\ \frac{dV_{r}}{dW_{0}}<0 & \mathrm{for}\;W_{0}>W_{c}^{*} \end{array}\;$$

The criticality of the binary mixture cannot provide a clear explanation of the change shown by Equation (59) since the criticality gives that $dV_{r}/dW_{0}=0$ and $d^{2}V_{r}/dW_{0}^{2}=0$ at $W_{0}=W_{c}$.

The change shown by Equation (59) is due to the $W_{0}$– (or $Q_{0}$–) dependence of the pre-factor B in Equation (44). Note that in the Spin-1 Ising model picture for the gel, $W_{0}$ corresponds to $Q_{0}$; increasing $W_{0}$ indicates increasing $Q_{0}$. From Equation (18), B is rewritten as:

(60) $$B=Q_{\mathrm{eq}}-Q_{0}-\phi_{\mathrm{eq}}\frac{\partial Q_{\mathrm{eq}}}{\partial \phi_{\mathrm{eq}}}\;.\;$$

Let us discuss the pre-factor B on the basis of the modified conventional Ising model (M-Ising model) picture given by Equation (56) for the binary mixture inside the gel. In terms of the order parameter $Q_{\mathrm{in}}=\langle \sigma \left(\vec{r}\right)\rangle$ of the M-Ising model, $Q_{\mathrm{eq}}$ is rewritten as:

(61) $$Q_{\mathrm{eq}}=\frac{n_{1}-n_{0}}{\Omega }=\frac{n_{0}+n_{1}}{\Omega }\frac{n_{1}-n_{0}}{n_{0}+n_{1}}=\left(1-\phi_{\mathrm{eq}}\right)\langle \sigma \left(\vec{r}\right)\rangle \;=\left(1-\phi_{\mathrm{eq}}\right)Q_{\mathrm{in}}\;,\;$$

and B is also rewritten as:

(62) $$B=Q_{\mathrm{in}}-Q_{0}-\phi_{\mathrm{eq}}\left(1-\phi_{\mathrm{eq}}\right)\frac{\partial Q_{\mathrm{in}}}{\partial \phi_{\mathrm{eq}}}\;$$

with

(63) $$\frac{\partial Q_{\mathrm{in}}}{\partial \phi_{\mathrm{eq}}}=\frac{2T_{\mathrm{eff}}}{1-\phi_{\mathrm{eq}}}\frac{\partial Q_{\mathrm{in}}}{\partial T_{\mathrm{eff}}}+\left(h+z_{0}J\right)\chi_{\mathrm{in}}\;,\;$$

where

(64) $$\chi_{\mathrm{in}}\equiv \frac{\partial Q_{\mathrm{in}}}{\partial h_{\mathrm{eff}}}\;$$

is the magnetic susceptibility of the conventional Ising model. Since the affinity between polyacrylamide and water is higher than that between polyacrylamide and isobutyric acid, we can expect that the ratio of isobutyric acid in the binary mixture inside the gel is smaller than that outside the gel; $Q_{\mathrm{in}}<Q_{o}$. Therefore, in the case where the third term on RHS of Equation (62) is negligible, B is always negative and $V_{r}$ is a monotonic decrease function of $W_{0}$; $\frac{dV_{r}}{dW_{0}}<0$. The swelling behaviors at T=35.0 and 45.0 ℃ in Figure 2 correspond to this case. As the binary mixture inside the gel approaches the critical point, the third term becomes non-negligible since the susceptibility $\chi_{\mathrm{in}}$ becomes very large. The increased susceptibility changes the sign of B when $h+z_{0}J<0$. This result allows that the sign of B (the sign of $dV_{r}/dW_{0}$) changes depending on the value of $Q_{0}$ (the value of $W_{0}$) since h is a function of $Q_{0}$; $h=h\left(Q_{0}\right)$. Hence, the presence of the maximum of $V_{r}$ at $W_{0}=W_{c}^{*}$ or the change expressed by Equation (59) is explained by the increase in $\chi_{\mathrm{in}}$ near the critical point of the binary mixture inside the gel.

The results of the theoretical analysis indicate that the criticality of the binary mixture induces the nonmonotonic swelling behavior of the gel, as shown in Figure 2.

The theoretical analysis developed in the present article is independent of details of the gel system. Therefore, the predictions derived from the theory are rather universal. For example, the shoulder in the swelling behavior near the critical temperature is expected to appear in the case of hydrophobic gels, as well as hydrophilic gels such as polyacrylamide gel reported in the present article.

For a single polymer in binary mixture, the theoretical result can be applicable if the chain length of the polymer is long enough and the polymer extends widely. Near the critical point of the binary mixture outside the extension region of the polymer chain, the composition of the binary mixture inside the extension region is insensitive to that outside the extension region. Therefore, composition change of the outside mixture does not change the size of the polymer extension. When the critical points of the outside mixture and of the inside mixture are near, increase of the “susceptibility” $\chi_{\mathrm{in}}$ of the inside mixture induces nonmonotonic swelling behavior of the polymer chain.

## 5. Materials and Methods

All the reagents except for water were purchased from Wako Pure Chemicals, Co., Ltd., Osaka, Japan Acrylamide (40 mg) and *N*, *N*′-methylenebisacrylamide (4 mg) were dissolved in 40 g of Milli-Q water on ice for 5 min. After bubbling of nitrogen gas in the solution, we added 50 μL of 15 w/v% ammonium persulfate and 1 mL of *N*, *N*, *N*′, *N*′- tetraethylenediamine to the solution and stirred the solution for 2 min. An aliquot of the solution was soaked up into a 10-cm-long Pyrex glass tube with an inner diameter of 2 mm with open ends and incubated to form a cylindrical gel at room temperature of 25 °C for 24 h. The glass tube was then immersed in Milli-Q water for a week. When the gel in the glass tube was immersed in MilliQ water, it came out from the glass tube spontaneously due to swelling. The gel could be completely peeled off from the glass tube by pushing the other end of the gel through water pressure using a syringe. The water was replaced with a fresh one several times to remove remaining monomers and impurities. The gel was cut into ca. 4 cm long pieces (the gel volume was ca. 0.12 mL) and further immersed in pure water at 26.4 °C. Then the lengths of the gels (*L*_0_) were measured with a cathetometer.

For the measurements of the cloud point of the binary mixture isobutyric acid water inside and outside the gels and the volume of the gels immersed in the binary mixture, isobutyric acid was distilled twice before measurements and mixed with Milli-Q water at weight fractions *W*_0_ of isobutyric acid in a range of up to 0.6. The cylindrical gels prepared as described above were immersed in 30 mL of isobutyric acid–water mixtures with different *W*_0_ values for 24 h in a water bath controlled at 45 °C. Then, the temperature of the solution containing a cylindrical gel was gradually lowered to 35, 28, 27, and 26.4 °C and incubated for 24 h. At each temperature, the length L of the gels was measured. The volume of the gel was calculated from the length of the gel by assuming isotropic swelling or shrinking, and the reduced volume was defined by $V_{r}={\left(L/L_{0}\right)}^{3}$. The temperature *T*_p_ at which the binary mixture inside and outside the gel became turbid was determined by decreasing the temperature of the bath at a rate of 0.01 K/min. To estimate the pore size of the gel network, we prepared a disk-like gel with a diameter of 25 mm and a height of 10 mm with the same preparation procedure as the above-mentioned cylindrical gels. The load (*P*)–indentation (*δ*) curve was measured using a laboratory-made apparatus consisting of a load cell (A&D LC4101-G600) and a stainless probe with a radius of *a* = 0.925 mm at 25 °C. The Young’s modulus *E* was estimated from the initial slope of the *P–δ* plot using the equation based on the Herz model: *P* = 2*Eaδ*/(1 − *ν*^2^) [20], where Poisson’s ratio *ν* was assumed to be 0.5.
